# Surgical treatment of equine colic - a retrospective study of 297 surgeries in Norway 2005–2011

**DOI:** 10.1186/1751-0147-56-38

**Published:** 2014-06-16

**Authors:** Bjørn H Wormstrand, Carl F Ihler, Ragnhild Diesen, Randi I Krontveit

**Affiliations:** 1 Department of Companion Animal Sciences, Faculty of Veterinary Medicine and Biosciences, Norwegian University of Life Sciences, P.O. Box 8146 Dep, 0033 Oslo, Norway; 2 Department of Food Safety & Aquatic Medicine, Faculty of Veterinary Medicine and Biosciences, Norwegian University of Life Sciences, P.O. Box 8146 Dep, 0033 Oslo, Norway; 3 Damtjernveien 78, 3175 Ramnes, Norway; 4 Current work address: Klinikken På Travparken AS, Travparkveien, 5111 Breistein, Norway

## Abstract

**Background:**

Colic, defined as pain originating from the abdomen, is a common condition in horses. Most of the cases resolve spontaneously or after medical treatment, but a few require surgical treatment. Surgical treatment of colic in horses is resource-demanding and expensive, and information on prognosis is therefore important for both owners and surgeons. In the present study, surgical cases in two equine hospitals in Norway between 2005 and 2011 were reviewed. The aim of the study was to describe associations between prognostic indicators, diagnoses and short term survival by use of random effects logistic regression.

**Results:**

In the present study, 162 out of 297 (54.5%) surgeries resulted in the horse being discharged from the hospital. Excluding cases euthanized during surgery, the overall short-term survival was 74.0% (162 out of 219 surgeries). Seventy-eight (26.3%) of the horses were euthanized during surgery, due to grave or poor prognosis. In univariable analyses, duration of colic signs, heart rate, capillary refill time, mucosal membrane appearance, intestinal sounds, affected gastrointestinal segment, hematocrit, intestinal resection, hospital and surgeon board-certification had *P*-value <0.20 and were assessed in multivariable analyses. Respiration rate, rectal temperature and lactate in blood also had univariable *P* <0.20, but were left out from multivariable analyses due to too high levels of missing values. A random effect of primary surgeon was included and breed, sex and age were tested in multivariable analyses as possible confounders; and hospital was included to control for hospital routine differences. In the final multivariable model the variables mucosal membrane appearance, affected gastrointestinal segment and surgeon board-certification significantly influenced survival. The random surgeon effect was not significant.

**Conclusions:**

The present study showed that prognostic parameters and diagnoses of surgical treatment of horses with colic in Norway are in accordance with reports from other parts of the world. The significant effect of board-certification of surgeon is not reported in previous studies. The general short-term survival rate was somewhat lower than reported in other studies, partly due to more horses being euthanized intraoperatively in the present study. This might be because of economical or animal welfare reasons.

## Background

Colic, defined as pain originating from the abdomen, is a common condition in horses. An incidence of 4.2-10.6 colic cases/100 horse-years is reported from the United States and Great Britain [1-4]. In Norway, the incidence of colic has been estimated to 4.8 cases/100 horse-years [5]. A Swedish study found an incidence of 0.91 colic cases/100 horse-years [6]. This study was, however, based on insurance claims. The majority of equine colic episodes resolve spontaneously or after medical treatment. On the other hand, displacements and especially strangulating lesions might prove fatal without surgery [7-9]. Colic surgery remains one of the most expensive and resource-demanding procedures performed in equine medicine, due to the staff and facilities necessary and the prognosis varies from guarded to excellent [10,11]. Information on the prognosis of surgical treatment is, therefore, important for both owners and surgeons in order to make the proper decision on treatment in the individual case.

There are a number of studies published on the survival of horses undergoing colic surgery, both for specific conditions and overall survival [9,12-18]. However, as pointed out by Mair *et al*. [19], the studies vary in design, mainly regarding inclusion criteria. Specific lesions [12,20], affected intestinal segment [21] and surgical treatment [10] are all used as inclusion criteria in various studies. Other studies include all colics, both medical and surgical [16-18]. Systematic studies on causes and treatment of colic are, therefore, essential in the continuous effort to improve clinical outcome. The aim of the present study was to describe associations between possible prognostic indicators, diagnoses and short term survival (STS), defined as a horse being discharged from the hospital, in surgical colic cases in two equine hospitals in Norway from 2005 to 2011.

## Methods

Case records of all horses undergoing laparotomy due to abdominal pain in two equine hospitals in Norway between 2005 and 2011 were reviewed, altogether 317 horses. All horses were admitted to the hospitals due to a perceived serious colic condition. Hospital 1 accepted only cases referred by a veterinarian in the period reviewed, while Hospital 2 also accepted horses without a veterinary examination beforehand. Of these, seven horses had lesions in organ systems other than the gastrointestinal tract diagnosed prior to surgery (two foals with rupture of the urinary bladder and five mares with uterus torsion) and were thus excluded from the study. Four horses were euthanized in the recovery stall due to fractures (n = 3) or femoral nerve paralysis (n = 1), and were also excluded from the study. Nine horses had a second laparotomy performed due to persisting colic and were omitted as well. Altogether 297 horses were included in the study population. The breed and age distribution of the included cases are presented in Table 1. Information retrieved from the records included duration of colic signs, transport time to the hospital (minutes), case history, results of preoperative clinical and laboratory examinations (hematocrit, blood lactate, total solids in blood), duration of anesthesia, surgical findings and procedures (enterotomy, resection), outcome, and duration of hospitalization. Also, primary surgeon, secondary surgeon, anesthetist, daytime vs. out of hours surgery, and surgeon specialist qualifications were included as variables. In cases where information on transport time to the hospital was missing, Google Map® was used to estimate the duration of transportation. Surgical treatment was in all cases chosen when clinical examinations showed bowel displacements on rectal examination, progressively worsening general condition or refractory pain. Duration of colic signs prior to surgery was recorded from information by the owner or referring veterinarian. Horses euthanized during surgery were omitted from statistical analysis of anesthesia time and whether enterotomy and resection of intestine were performed or not.

**Table 1:** Breed and age of 297 horses surgically treated for colic in Norway 2005 - 2011

**Breed**	**n**	Median age*	Range*
Warmblood	101	9.5	0 -18
Standardbred	46	4	0 - 20
Pony Breeds	45	10	1 - 20
Norwegian Coldblooded Trotter	42	5.5	0 - 30
Thoroughbred	18	4	0 - 11
Icelandic Horse	16	7.5	0.5-17
Other Breeds	28	8	0 - 18
Unknown	1	16	
Total	297	7	0 - 30

*Given in years. 0 = Foals 1–2 days of age.

The results of the last physical examination prior to surgery were defined as preoperative clinical variables. Thirty horses also had the total solids, referring to proteins and lipids, of abdominal fluid measured. Lactate measurements were available at Hospital 1 for the whole period, and at hospital 2 from 2010 and onwards. At Hospital 1 an ABL-800 FLEX® (Radiometer Medical, Brønshøj, Denmark) was used for the lactate analysis, and at Hospital 2 a handheld Accutrend® (Labservis LTD, Baku, Azerbaijan) was used. Total solids in plasma and abdominal fluid were measured by a refractometer (ATAGO CO., LTD, Tokyo, Japan). As lactate had almost 50% missing values and was measured with two different methods, this variable was left out from the analyses. An overview of the variables with total number of observations and number and percentage missing observations of each variable is presented in Table 2.

**Table 2:** Overview of observed variables with number of horses (n) and missing observations in each variable in a retrospective study of 297 colic surgeries in Norway 2005 - 2011

**Variable**	**n**	**Missing (%)**
Prior to admission		
Duration of signs (hours)	271	26 (8.8)
Transport time (minutes)	292	5 (1.7)
Preoperative variables		
Heart rate (beats\minute)	271	26 (8.8)
Respiration rate (elevated\not elevated)	151	146 (49.2)
Rectal temperature (˚celsius)	167	130 (43.8)
Capillary refill time (seconds)	227	70 (23.6)
Mucosal membranes (appearance)	268	29 (9.8)
Normal, pale, toxic, cyanotic or sligthly abnormal		
Rectal examination	235	62 (20.9)
Normal, impaction of large intestine, displaced colon, tympanic colon or distended small intestine		
Borborygmia	237	60 (20.2)
None, reduced, normal, increased or tympanic		
Nasogastric reflux (positive\negative)	155	142 (47.8)
Hematocrit	251	46 (15.5)
Total solids in plasma (gram\litre)	246	51 (17.2)
Lactate in blood (mmol\litre)	153	144 (48.5)
Intraoperative variables		
Anesthesia duration (minutes)*	187	32 (10.7)
Enterotomy (performed\not performed)*	219	0 (0.0)
Intestinal resection (performed\not performed)*	219	0 (0.0)
Anesthetist	244	53 (17.8)
Primary surgeon	286	11 (3.7)
Secondary surgeon	210	87 (29.3)
Surgeon board-certification (yes\no)	294	3 (1.0)
Daytime vs. out-of-hours	283	14 (4.7)
Postoperative variables		
Hospitalisation duration (days)*	218	1 (0.4)

*Excludes cases that were euthanized during surgery (n = 78).

All laparotomies were performed under general anesthesia in dorsal recumbency. Enterotomy and resection of bowel were classified as performed/not performed. Surgeons worked in a team of two for the majority of surgeries. All surgeons serving as primary surgeons had several years of experience with laparotomies in horses. Eleven different surgeons were serving as primary surgeon in the period reviewed, with 30 different surgeons serving as assistant surgeons. Two surgeons were board-certified by the American College of Veterinary Surgeons (ACVS) and one by the European College of Veterinary Surgeons (ECVS), all three working at Hospital 1. Twenty six different anesthetists were involved. Diagnoses were based on surgical exploration, with the exception of horses euthanized during surgery where a final diagnosis was made at post mortem examination.

Both the participating hospitals are located in Oslo, and mainly serve the equine population in the south-eastern part of Norway, although cases are referred from all over the country. The surgical team performing colic surgeries at both hospitals consists of two surgeons and a nurse assisting. At Hospital 1 a veterinarian or a nurse specialized in anesthesia are responsible for the anesthesia, while at Hospital 2 a nurse or intern is handling anesthesia.

Pre-operative treatment at both hospitals was constantly adjusted according to recent literature in the period reviewed, but in general consisted of penicillin 22.000 IE/kg body weight (bwt) intravenously (iv.), gentamycin 6.6 mg/kg bwt iv., flunixin meglumine 1.1 mg/kg bwt iv., fluid therapy according to need and further analgetics if needed (α-agonists and butorphanol iv.). Postoperatively, antibiotics (procaine penicillin 22.000 IE/bwt intramuscularly (im.) twice a day and gentamycin 6.6 mg/kg bwt iv. once a day) were continued for 5–10 days and flunixin meglumine for at least 3 days, longer if needed. Sucralfate (8 mg/kg bwt perorally (po.) three times a day) were given as prophylaxis against gastric ulcers until the horse was back on normal feed ration, and omeprazole (4 mg/kg bwt po. once a day) if gastric ulcers were confirmed. In cases involving resection of intestine, strangulated small intestine or ileus was treated with lidocaine as a bolus of 1.3 mg/kg bwt iv. followed by constant rate intravenous infusion of 0.05 mg/kg bwt/min iv. for 24–48 hours postoperatively. Clinical status was taken every third hour 24–48 hours postoperatively, or longer if needed. Hematocrit, total solids in blood and blood gas analysis were done 1–2 times a day, or more often if indicated. A nasogastric tube passed as needed if ileus or discomfort occurred.

### Statistical analysis

The main outcome variable was short-term survival (STS) defined as a horse being discharged from the hospital, or not. The outcome variable was thus a dichotomous variable and logistic regression was applied. Variables (respiration rate, lactate in blood, reflux) with more than 25% missing observations were omitted from the analyses. The variables were first tested unconditionally with univariable logistic regression. Continuous variables were evaluated for linearity by lowess curves and by adding the quadratic term of the variable. Continuous variables showing a non-linear relationship with the outcome variable were categorized into biologically plausible categories. Thus, the variables duration of signs, heart rate, capillary refill time, hematocrit, anesthesia duration and hospitalization time displayed a non-linear relationship with the outcome and were categorized. Heart rate was classified as normal or elevated (>44 beats per minute), capillary refill time classified as less than 2 seconds, between 2 and 3 seconds, and more that 3 seconds, and hematocrit was classified as normal (0.32-0.42), low (<0.32) or increased (>0.42). The variable duration of signs was grouped into four categories based on percentiles. Anesthesia duration was classified as less than 120 min, more than 120 min or as euthanized intraoperative. Variables were then screened for colinearity by pair wise correlations for continuous variables and by Goodman and Kruskal’s gamma for dichotomous and ordinal variables [22,23]. Associations > 0.7 or < -0.7 were considered evidence of colinearity. The variables with a univariable *P*-value ≤ 0.20 from the likelihood ratio test (LRT) and the multiple Wald test were selected for further multivariable analysis, provided that there was no colinearity between them. When colinearity was detected, the variable with the strongest univariable *P*-value and fewest missing data was selected for further analysis.

There were 11 primary surgeons with an average of 26 records per surgeon, and a random effect of surgeon was used in the analyses. As only Hospital 1 had board-certified surgeons available in the revised period, the statistical analysis of this parameter was also done with a reduced data set, containing only surgeries performed in Hospital 1. A multivariable random effects logistic regression model with outcome survival yes/no was constructed by manual forward selection by offering variables selected from the univariable analyses one-at-a-time to the model by ascending univariable *P*-value. Variables were retained in the model when the *P*-value of the LRT and multiple Wald test was < 0.05. Potential confounding and intervening variables were evaluated based on both a tentative causal diagram and changes in effects during model building. All possible two-way interactions between statistically significant variables in the model were tested by adding interaction terms to the model. Due to the large number of interactions tested, a more restrictive *P*-value was applied. Interaction terms were retained if the *P*-value of the LRT was < 0.01. The LRT was used to evaluate the significance of the random surgeon effect in the models with and without random surgeon effect, but containing the same fixed effects. The LRT was considered significant at *P* = 0.05 and one-sided test.

The multiple Wald test and LRT were used to evaluate differences between categories of categorical predictors. The Stata command lincom was used to conduct contrasts among each category of categorical predictors.

From the final multivariable random effects logistic regression model the between surgeon variance (σ^2^_surgeon_) was estimated. The intraclass correlation coefficient (ICC) calculated by the latent variable approach, assuming that horse level variance (σ^2^) is the constant π^2^/3 was calculated using.

$$\mathrm{ICC}={\sigma^{2}}_{\mathrm{surgeon}}/\sigma^{2+}{\sigma^{2}}_{\mathrm{surgeon}}$$

To evaluate and assess the fit of the final multi-level model the residuals at the horse level were estimated and the residuals at the surgeon level were estimated and evaluated by plotting of residuals against both predicted values and against fitted values to evaluate homoscedasticity and normality [22].

The software package Stata 12® (Stata Corporation, 4905 Lakeway Drive, College Station, TX 77845, USA) was used for all analyses.

## Results

### Descriptive statistics

Out of the 297 horses there were 132 (44.4%) geldings, 111 (37.4%) females and 53 (17.8%) intact males. Sex was not recorded in one case. Median age was 7 years.

A total of 179 surgeries were performed at Hospital 1 and 118 surgeries were performed at Hospital 2. Of the 316 surgeries performed, 162 (54.5%) resulted in the horse being discharged from the hospital. Excluding the 78 cases euthanized during surgery, the overall short-term survival was 74.0% (162 of 219 surgeries). The occurrence of each diagnosis and the corresponding short-term survival are presented in Table 3.

**Table 3:** Lesions, frequency and short-term survival rates of 297 colic surgeries in Norway 2005 - 2011

**Lesion**	**n (%)**	**Discharged from hospital (%)**	**Euthanized during surgery (%)**	**Euthanized after surgery (%)**
**Ventricle**	**3 (1.0)**	**1 (33.3)**	**2 (66.7)**	**0 (0.0)**
Impaction	1 (0.3)	1	0	0
Rupture	2 (0.7)	0	2	0
**Small intestine**	**96 (32.3)**	**46 (47.9)**	**34 (35.4)**	**16 (16.7)**
Impaction	11 (3.7)	10	1	0
Impaction by ascarides	4 (1.3)	1	1	2
Volvulus	9 (3.0)	4	3	2
Incarceration in epiploic foramen	18 (6.1)	5	9	4
Incarceration in mesenteric rent	7 (2.4)	2	2	3
Intussusception	5 (1.7)	3	2	0
Incarceration in inguinal hernia\rupture	12 (4.0)	9	3	0
Verminous endarteritis	1 (0.3)	0	1	0
Infarct	7 (2.4)	1	5	1
Enteritis	14 (4.7)	8	4	2
Rupture	2 (0.7)	0	2	0
Strangulation by pedunculated lipoma	3 (1.0)	0	1	2
Ileus without obstruction	3 (1.0)	3	0	0
**Cecum**	**26 (8.8)**	**7 (26.9)**	**9 (34.6)**	**10 (38.5)**
Impaction	12 (4.0)	2	6	4
Displacement	8 (2.7)	4	1	3
Tympany	2 (0.7)	1	0	1
Intussusception	4 (1.3)	0	2	2
**Large colon**	**135 (45.5)**	**90 (66.7)**	**21 (15.6)**	**24 (17.7)**
Impaction	23 (7.7)	20	0	3
Volvulus	37 (12.5)	14	13	10
Left dorsal displacement	20 (6.7)	16	3	1
Right dorsal displacement	33 (11.1)	27	2	4
Other displacements including retroflexion of the pelvic flexure	10 (3.4)	8	1	1
Rupture	2 (0.7)	0	2	0
Colitis	5 (1.7)	1	0	4
Tympany	3 (1.0)	3	0	0
Intramural abscess	1 (0.3)	1	0	0
Incarceration in diafragmatic rent	1 (0.3)	0	0	1
**Small colon**	**15 (5.1)**	**8 (53.3)**	**4 (26.7)**	**3 (20.0)**
Impaction	12 (4.0)	6	3	3
Meconium retention	3 (1.0)	2	1	0
**Peritoneal cavity**	**6 (2.0)**	**1 (16.7)**	**5 (83.3)**	**0 (0.0)**
Peritonitis	3 (1.0)	1	2	0
Adhesions	3 (1.0)	0	3	0
**Miscellaneous**	**16 (5.4)**	**9 (56.2)**	**3 (18.8)**	**4 (25.0)**
Faecalith obstruction**	8 (2.7)	4	2	2
Strictures*	5 (1.7)	4	0	1
Ovarian neoplasia involving small intestine	1 (0.3)	1	0	0
Unknown	2 (0.7)	0	1	1
**Total**	**297**	**162 (54.5)**	**78 (26.3)**	**57 (19.2)**

*Includes two cases with strictures in the pelvic flexure and three with strictures in the jejunum.

**Four in the small colon, two in the pelvic flexure, one in the transverse colon, one in the jejunum.

Descriptive statistics and results with odds ratio (OR) and *P*-values for variables with less than 25% missing values, and *P*-value below 0.2 from the univariable analyses are presented in Table 4. Of the 235 cases that had a reported rectal examination, 12 cases (5.1%) had normal findings, 42 (17.9%) findings corresponding to impaction of large intestine, 66 (28.1%) findings suggesting displacement of large intestine, 76 (32.3%) distended small intestine and 39 (16.6%) tympany of large intestine. Of the 155 cases with information on nasogastric intubation, 87 cases (56.1%) had reflux of gastric content when a nasogastric tube was passed. Only 29 cases had information on solid content in peritoneal fluid and this variable was excluded from statistical analysis. When excluding cases euthanized intraoperatively, mean anesthesia time was 134 minutes (range 45–360 minutes).

**Table 4:** Variables showing p < 0.20 in univariable analysis in 297 colic surgeries in Norway 2005–2011

**Variable**	**Mean (range)/ number (%)**	**OR**	**p-value**
Hospital			
Hospital 1	179 (60.2)	1.00	
Hospital 2	118 (39.7)	0.65	0.146
Duration of signs (hours)			
0 - 8	53 (19.5)	1.00	
8 - 16	79 (29.0)	1.62	0.191
16 - 30	68 (15.0)	1.42	0.364
>30	72 (26.5)	0.75	0.441
Transport time (minutes)	88.6 (0–900)	1.003	0.032
Preoperative variables			
Heart rate			
Normal (≤44 bpm)	62 (22.9)	1.00	
Increased (>44 bpm)	209 (77.1)	0.52	0.043
Capillary refill time			
< 2 seconds	96 (42.3)	1.00	
2-3 seconds	110 (48.5)	1.06	0.843
> 3 seconds	21 (9.3)	0.29	0.029
Mucosal membranes			
Normal	108 (40.2)	1.00	
Slightly abnormal	81 (30.2)	0.40	0.005
Pale	54 (20.1)	0.31	0.001
Cyanotic	10 (3.7)	0.14	0.024
Toxic	15 (5.6)	0.40	0.514
Intestinal sounds			
Normal	22 (9.3)	1.00	
Reduced	110 (46.4)	0.55	0.297
Abscent	78 (32.9)	0.29	0.033
Tympanic	19 (8.0)	0.64	0.539
Increased	8 (3.4)	0.19	0.070
Hematocrit			
Normal (0.32 - 0.42)	120 (47.8)	1.00	
Low (<0.32)	37 (14.7)	0.92	0.834
Increased (>0.42)	94 (37.5)	0.50	0.021
Intraoperative variables			
Board-certified surgeon (yes\no)			
No	201 (68.4)	1.00	
Yes	93 (31.6)	2.14	0.004
Anesthetist (continuous)	8 (1–26)	0.97	0.181
Affected GI-segment			
Small intestine	101 (34.0)	1.00	
Cecum	25 (8.4)	0.42	0.081
Large colon	141 (47.5)	2.02	0.011
Small colon	19 (6.4)	1.10	0.851
Other	10 (3.4)	0.67	0.557
Intestinal resection (yes\no)*			
No	198 (90.4)	1.00	
Yes	21 (9.6)	0.42	0.064

*Cases euthanized during surgery left out.

Variables with >25% missing data omitted.

Seventy-eight (26.3%) of the horses were euthanized due to grave prognosis because of rupture/perforation of intestine (n = 10) or perceived poorer prognosis due to non-viable intestine (n = 68). Of the 219 cases brought into recovery, 97 (44.3%) had an enterotomy performed and 21 (9.6%) had intestinal resection and anastomosis performed.

The variables that had univariable *P*-value ≤ 0.20 and thus were eligible for inclusion in the multivariable analyses were: duration of colic signs, heart rate, mucus membrane appearance, capillary refill time (CRT), hematocrit, intestinal sounds, affected gastrointestinal segment, intestinal resection, duration of hospitalization, primary surgeon, and whether the primary surgeon was a board-certified specialist or not. Breed, sex and age were included as potential confounders, and hospital was included as a fixed effect to control for hospital routine differences.

### Multivariable analyses

Results with OR, *P*-values, and 95% confidence intervals from the final multivariable model are presented in Table 5. The variables affected GI segment, mucosal membranes, and specialist surgeon were significant. Lesions in the large colon doubled the odds of survival when compared with lesions in small intestine which was the baseline; lesions in the cecum and small colon gave approximately halved odds of survival. All categories of abnormal mucosal membrane appearance, especially cyanotic, reduced the odds of survival when compared to the normal (baseline) appearance. If the surgeon was specialist, this significantly increased the survival by almost 2.5, while the effect of hospital was non-significant when including the variable specialist surgeon. Breed, sex and age did not show any confounding effects. Both effect of anesthetist and daytime vs. out-of –hours surgery was tested and neither of these variables showed any significant effect. Significant interactions were not found. As a sensitivity analysis, the model was applied on a reduced dataset containing only records from Hospital 1 and this gave almost identical results (data not shown).

**Table 5:** Final multivariable model of variables significant for survival in 297 colic surgeries in Norway 2005 - 2011

	**OR, survival**	**p**	**95% CI**
Affected GI-segment		0.007^a^	
Small intestine	1.00		
Cecum	0.44	0.145	0.15 - 1.33
Large colon	2.03	0.021	1.11 - 3.71
Small colon	0.94	0.906	0.32 - 2.74
Ventricle and other	0.90	0.900	0.18 - 4.43
Mucosal membranes		<0.001^a^	
Normal	1.00		
Abnormal	0.42	0.009	0.21 - 0.81
Pale	0.32	0.003	0.15 - 0.81
Cyanotic	0.18	0.028	0.04 - 0.84
Toxic	0.59	0.396	0.18 - 1.99
Surgeon			
Not board-certified	1.00		
Board-certified (ECVS\ACVS)	2.47	0.013	1.21 - 5.06

^a^Likelihood ratio test.

When building the multivariable model, the random surgeon effect was moderate at 6%, but significant (ICC 0.06, p = 0.032) until adding the variable board-certified surgeon or the variable hospital. Then the random surgeon effect became negligible at 0.5% and non-significant (ICC 0.005, *P* = 0.225).

The surgeon level residuals from the random part of the final model showed no evidence of heteroscedasticity or lack of normality. No extreme values were found in the horse level residuals.

## Discussion

The overall STS of 54.8% in the present study is lower than reported in other similar studies, which report an overall survival rate from 48.0% to 85.0% [9-11,13,14,24]. Findings regarding intestinal segment and type of lesion are in accordance with other studies [11].

In the present study 26.3% of the 297 surgeries resulted in the horse being euthanized during surgery. This is considerably higher than the 13.0% intraoperative mortality reported by Mair & Smith [10] and Santschi *et al.*[14] and the 8.2% reported by Proudman *et al.*[25]. In a Canadian study where horses were referred over substantial distances, however, the intraoperative mortality was similar (26.5%) [16]. One reason for high number of intraoperative euthanasia in the present study might be related to attitudes towards animal welfare as Norwegian owners and veterinarians might be reluctant to proceed with procedures resulting in extended periods of discomfort. This is, however, not an easily assessed factor. Furthermore, financial limits might be a reason for intraoperative euthanasia, especially when prognosis is poor. Norway is a high-cost country and owners may be reluctant to go on with treatment of cases with reduced prognosis and longer recovery period. When a serious condition in need of for example intestinal resection was found during surgery, the surgeon usually called the owner and provided the owner with this information. The owner then made the decision of whether to go ahead with the surgical procedure. The present study did not investigate the reasons why owners elected to have the horse euthanized during surgery.

Fifty seven (19.1%) of the 297 horses that underwent surgery were euthanized in the postoperative period. The reasons for euthanization were not documented for each case, but in general ileus, persisting colic signs, laminitis and animal welfare concerns were common reasons for euthanasia. Economical limitations are also involved, as prolonged intensive care rapidly becomes expensive.

STS for large colon volvulus in the present study was low, with 14 out of 37 cases discharged (STS 37.5%), compared to the STS of 70.7% reported by Suthers *et al*. [20]. Also, incarceration of small intestine in the epiploic foramen had a low STS (5 out of 18 cases discharged, 27.8%) compared to a multicenter study [26]. These are both rapidly progressing conditions often leading to severely compromised and sometimes non-viable intestine, requiring resection and anastomosis of intestine. The intraoperative euthanasia of respectively 35.1% and 50.0% as well as the low STS for such cases in the present study suggests that earlier surgical treatment is essential in these kinds of cases. Many studies with reports of an exceptionally high STS for such lesions originate from horse-dense areas where owners often are professional and veterinarians often exclusively work in equine practice [27]. Most likely, this will lead to earlier referral for surgical treatment. This is in contrast to Norway which have a rather dispersed equine population and where veterinarians on call commonly are general practitioners. The decision of performing surgery and euthanasia on severely ill patients is ultimately an owner decision which also influences the intraoperative mortality if owners in different regions have different attitudes. Precise diagnosis is important to provide an accurate prognosis, and this is difficult to obtain preoperatively in most cases of colic. It is therefore likely that the decision of choosing euthanasia is easier made with the more precise diagnosis obtained during surgery. Implementation of peritoneal fluid analysis in the preoperative examination of colic cases will likely contribute to a more accurate prognosis, but in the present study, examination of total protein in peritoneal fluid was only performed in 30 horses.

Impaction of the cecum had a low STS in the present study, with 2 out of 12 cases discharged (STS 16.7%), which is low compared to the 65% reported by Smith *et al*. [28]. One study reported a STS for cecal impactions of 75.5% [29]. Cecum impactions are challenging and complicated cases of colic and often treated medically for an extended period of time before decision on surgery is taken. The low STS of cecum impactions in the present study might suggest that surgical intervention is indicated at an earlier stage. The risk of intestinal rupture during surgical manipulation is increased for cecal impactions compared to impactions in other parts of the intestine [29]; this also contributes to the reduced survival for this type of lesion.

The distribution of different lesions requiring surgical treatment in the present study is similar to the study by Mair and Smith [10], although some exceptions do exist. The number of cases with pendunculated lipomas was very low in the present material (n = 3). This is most likely due to a relatively young population with a median age of 7 years, while Mair and Smith [10] reported a median age of 11 years. Likewise, anterior enteritis and grass sickness are rare diseases in Norway, making colic patients with distended small intestine loops usually surgical patients. On the other hand, the present study included 16 cases with enteritis undergoing surgery. This could indicate that clinicians are not routinely faced with these diseases.

The cardiovascular parameters were significantly associated with survival in the present study. This is in accordance with most studies published on prognostic factors in treatment of both surgical and medical equine colic [8,11,17,24,25].

The rectal examination findings were not found to have a significant effect on survival in these surgical colic cases. Findings on rectal examination is one of the important criteria in the decision for choosing surgical treatment, and is thus biased towards abnormal findings.

Affected intestinal segment did show significantly influence on survival. Lesions in the large colon doubled the odds of survival compared to small intestine. This is in accordance with other studies [9,10,17], and related to the prevalence of lesions in the different segments of intestine, as strangulating lesions relatively occur more often in the small intestine.

Although there were reduced odds for survival when the duration of anesthesia exceeded 120 minutes, this was however not statistically significant to survival in the univariable analysis. This is most likely a reflection of the fact that severe conditions require more complicated procedures and thus a longer period of anesthesia, without the prolonged anesthesia itself being cause of increased mortality.

The random effect of primary surgeon, and the fixed effects of surgeon specialist and hospital all were significant to the outcome in univariable analysis. In the multivariable model, however, the effect of hospital and the random effects primary surgeon were not significant when surgeon specialist was added as a fixed effect. Although all surgeons serving as primary surgeons in this study had long experience of abdominal surgery, the training and requirements to become a board-certified surgeon are likely to improve the techniques and skills of the surgeon, and thus increase survival. Likewise, differences in intraoperative procedures, other than the strictly surgical technique, and post-operative treatment could be of influence, as this most often is the responsibility of the primary surgeon. Only Hospital 1 had board-certified surgeons available during the period investigated, and the effect was significant also when including only cases from this hospital.

Missing data are common in observational studies, and in the present study there were varying degrees of missing observations for some variables, some as high as 50%. This might be related to the fact that most colic cases are acute and sometimes dramatic, and record keeping might not be given a high level of priority. High numbers of missing data can also be a reflection of the attending clinicians’ attitudes towards the importance of different parameters. For instance, parameters such as heart rate, mucosal membranes, hematocrit and intestinal sounds, long established as good prognostics in equine colic patients, usually were recorded. More surprising was that 47.8% of the cases are missing information on reflux of gastric content, also a recognized prognostic parameter. This might be because results retrieved during clinical examination sometimes are not recorded if not providing decisive information. Statistical programs for building regression models work on the basis of complete sets of observations, and even a relatively low overall percentage of missing values can result in a substantial reduction in statistical power and hence fail to detect associations that are in fact present. Although the present study identified a number of significant variables related to STS of surgically treated colic patients, more complete records would have provided a better basis for multivariable analyses. Missing data can if not occurring at random, introduce bias and the analysis might result in associations that are severely biased and not valid. For instance, the blood lactate level in blood is often recognized as a significant variable regarding STS in surgical treatment of colic, but had almost 50% missing values in the present study. It might be that the patients missing the blood lactate parameters were unequally distributed across the outcome variable and other predictor variables than the patients where the blood lactate were recorded. Thus, including this variable in a multivariable model might bias the results of the model. Even if blood lactate was not included in the present study, other studies have identified colinearity with other circulatory variables such as heart rate, mucosal membrane appearance and hematocrit [24].

Retrospective studies on the survival and prognosis after surgical treatment of equine colic are regularly published [11]. Many of these studies only include univariable analysis of prognostic parameters [10,11]. A strength of the present study was that explanatory variables were assessed by multivariable regression analysis to be able to investigate effects of several factors simultaneously, evaluate and control for confounders, and detect intervening factors as well as interactions. In addition, the inclusion of a random surgeon effect controlled for and gave an estimate of the effect surgeon had on STS.

## Conclusions

The present findings regarding surgical treatment of equine colic patients in Norway are in many aspects similar to those reported from other parts of the world, although the survival rate is lower than in similar studies. In addition, the present study showed an increased survival if the surgeon was board-certified. Studies on long-term survival have become available in recent years, and would be a logic next step for further research, although long-term follow up often is practically challenging.

## Competing interests

The authors declare that they have no competing interests.

## Authors’ contributions

BW collected the data from Hospital 1, participated in the design of the study and drafted the manuscript. CI helped draft the manuscript and participated in the design of the study. RD collected the data from Hospital 2 and participated in the design of the study. RK performed the statistical analysis and helped draft the manuscript. All authors read and approved the final manuscript.
